# Crystal structure of X‐prolyl aminopeptidase from*Caenorhabditis elegans*: A cytosolic enzyme with a di‐nuclear active site

**DOI:** 10.1016/j.fob.2015.03.013

**Published:** 2015-04-02

**Authors:** Shalini Iyer, Penelope J. La-Borde, Karl A.P. Payne, Mark R. Parsons, Anthony J. Turner, R. Elwyn Isaac, K. Ravi Acharya

**Affiliations:** ^1^ Department of Biology and Biochemistry, University of Bath, Claverton Down, Bath BA2 7AY, UK; ^2^ Faculty of Biological Sciences, Clarendon Way, University of Leeds, Leeds LS2 9JT, UK; ^3^ School of Biosciences, University of Birmingham, Birmingham B15 2TT, UK; ^4^ Faculty of Life Sciences, University of Manchester, Manchester M13 9PL, UK; ^5^ Sevenoaks School, Sevenoaks TN13 1HU, UK

**Keywords:** APP1, aminopeptidase P1, CCP4, computational collaborative project 4, ICP-AES, inductively coupled plasma atomic emission spectroscopy, ICP-MS, inductively coupled plasma mass spectrometry, MAP, methionine aminopeptidase, NMR, nuclear magnetic resonance, PCR, polymerase chain reaction, PEG3350, polyethylene glycol 3350, rmsd, root mean square deviation, XPNPEP, X-prolyl aminopeptidase, Apstatin, Di-nuclear active site, Protease inhibitor, X-ray crystallography, X-prolyl aminopeptidase, Zinc metalloprotease

## Abstract

Eukaryotic aminopeptidase P1 (APP1), also known as X‐prolyl aminopeptidase (XPNPEP1) in human tissues, is a cytosolic exopeptidase that preferentially removes amino acids from the N‐terminus of peptides possessing a penultimate N‐terminal proline residue. The enzyme has an important role in the catabolism of proline containing peptides since peptide bonds adjacent to the imino acid proline are resistant to cleavage by most peptidases. We show that recombinant and catalytically active*Caenorhabditis elegans* APP‐1 is a dimer that uses dinuclear zinc at the active site and, for the first time, we provide structural information for a eukaryotic APP‐1 in complex with the inhibitor, apstatin. Our analysis reveals that*C. elegans* APP‐1 shares similar mode of substrate binding and a common catalytic mechanism with other known X‐prolyl aminopeptidases.

## Abstract 2

- We present the crystal structure of*C. elegans* APP‐1 both in bound and unbound forms.
- We show*C. elegans* APP‐1 uses dinuclear zinc at the active site.
- We confirm that*C. elegans* APP‐1 is biological dimer.
- Our analysis reveals that*C. elegans* APP‐1 shares a common catalytic mechanism with other X‐prolyl aminopeptidases.

## 1 Introduction

Metallopeptidases make up around a third of all known proteolytic enzymes and use an activated water molecule coordinated to a divalent metal ion as the catalytic nucleophile for peptide bond hydrolysis[1, 2]. The active sites of most metalloproteases contain a single metal ion; however enzymes belonging to the M24 family possess two metal ions that co‐catalytically activate a bridging water molecule[2]. M24 metallopeptidases are also characterised by having a common protein fold in their catalytic domain called the ‘'pitta‐bread’ fold that was first described in*Pseudomonas putida* creatinase[3]. The family is split into two subfamilies; subfamily M24A contains the methionine aminopeptidases (MetAP), whilst subfamily M24B includes two proline‐specific peptidases, X‐pro dipeptidase (prolidase) and X‐prolyl aminopeptidase (aminopeptidase P, APP)[2]. Proline is different from the other common proteinogenic amino acids in that it is an imino acid where the side chain is part of a five‐membered ring that includes both the Cα and the amino nitrogen atom. The resulting ring structure restricts free rotation around the Cα–N bond and the presence of a secondary amine means that the nitrogen cannot participate in hydrogen bonds with carbonyls[4]. As a consequence of the conformational rigidity conferred by the cyclic side‐chain, peptide bonds involving proline are often resistant to hydrolysis by peptidases[5, 6]. The protection conferred by the positioning of proline in a peptide sequence has often been exploited in nature to stabilize biologically active peptides from unwanted degradation and inactivation. However, there is a need for proline‐specific peptidases that can be used strategically to terminate the activity of peptide hormones (e.g. bradykinin) and for the catabolism of peptides derived from proline‐rich proteins, such as collagen[7, 8, 9].

Of the proline‐specific peptidases only APP and prolidase are capable of cleaving substrates at the N‐terminus where proline is in the P1′ position[10]. APP specifically cleaves the N‐terminal Xaa‐Pro peptide bond from oligopeptides and is distinct from prolidase, which acts only on dipeptides. APP activity, whilst not essential in*Escherichia coli* [11], is wide spread among bacterial genomes, including the minimal genome of*Haemophilus influenza*. In eukaryotes, there are three APP homologues, a soluble cytosolic form known as APP‐1, a membrane bound form called APP‐2 and a mitochondrial APP‐3[12, 13, 14, 15]. APP‐3 is evolutionarily distinct from the other APP enzymes, sharing just 12% and 16% sequence identity with APP‐1 and APP‐2, respectively. Within animals, APP‐1 is distributed widely among various tissues and cell types. In mammals, APP‐1 has been found in every tissue examined with strongest expression levels in the intestine, testis, kidney and brain. Similarly, Western blot analysis using a specific antibody showed APP‐1 expression in the gut, brain, testes and ovaries of adult*Drosophila melanogaster* [16] with RNA levels highest in the adult midgut and renal tubules[17]. In the free‐living nematode,*Caenorhabditis elegans*, the expression of an APP‐1‐GFP fusion protein was limited to the intestine, indicating a more restricted tissue distribution[18].

One of the proposed roles for APP‐1 is in the breakdown of imino‐peptides generated during protein catabolism. A deficiency in APP‐1 activity in human and mouse results in a large increase in proline‐containing oligopeptides in the urine (peptiduria) and severe developmental retardation, possibly from the failure to clear peptides from nerve cells[19, 20]. In the malaria parasite,*Plasmodium falciparum*, APP‐1 is localised to the food vacuole of the intraerythrocytic stage where haemoglobin is digested[21]. The ectoenzyme APP‐2 has a more specific role in the metabolic inactivation of signalling peptides (e.g. bradykinin and substance P) at cell surfaces[22, 23]. APP‐3 is involved in proteolytic processing and stabilization of mitochondrial proteins in yeast, but the substrates and metabolic role of the mammalian enzyme have not yet been precisely defined[24]. However, APP‐3 is physiologically important since mutations in*XPNPEP3*, the human APP‐3 gene, result in autosomal recessive kidney disease nephronophthisis[15].

*E. coli* APP‐1 and human cytosolic APP‐1, to date, are the only two enzymes to have had their three‐dimensional structures determined by X‐ray crystallography[25, 26, 27, 28, 29]. Here, we report the crystal structures of native as well as apstatin‐bound*C. elegans* APP‐1. Apstatin is a selective inhibitor of aminopeptidase P, designed specifically to test its effect on bradykinin degradation in rat lung[30]. To our knowledge this is the first structure of an inhibitor‐bound eukaryotic aminopeptidase P. Although a simple metazoan,*C. elegans* shares many essential molecular pathways with higher animals and has been exploited extensively as a powerful model to study molecular mechanisms linked to human diseases[31, 32]. In addition the nematode has been the subject of genome scale effort to determine three‐dimensional protein structures and new protein folds by X‐ray crystallography and NMR[33]. A better understanding of the structure/function of APP‐1 in*C. elegans* will help shed light on evolutionarily conserved functions of APP‐1 in other animals, including humans. This specialist aminopeptidase is also of interest in the development of safer methods for the treatment of diseases caused or transmitted by parasitic nematodes and arthropods; for example, APP‐1 has recently been proposed as a target for the development of anti‐malarial protease inhibitors.

Unlike the bacterial enzyme, eukaryotic APPs are larger (typically 620–650 residues long compared to 450), and whilst they share some clear primary sequence homology in the C‐terminal catalytic domain, there is very little, if any, sequence homology in the N‐terminal regions of these proteins. As such*E. coli* possesses relatively low sequence identity with human APP‐1 and APP‐2 (11% and 12% respectively), whilst*C. elegans* APP‐1 possesses 40% and 30% identity (58% and 48% similarity) with the two human homologues respectively. The structure of*C. elegans* APP‐1 bound to the inhibitor, apstatin, provides a better basis for the future development of novel approaches for parasite control. It would also help to provide a more accurate modelling of human APP‐2, a potential drug target for prevention of myocardial infarction[34].

## 2 Results

### 2.1 Analytical ultracentrifugation

**Figure 1:**
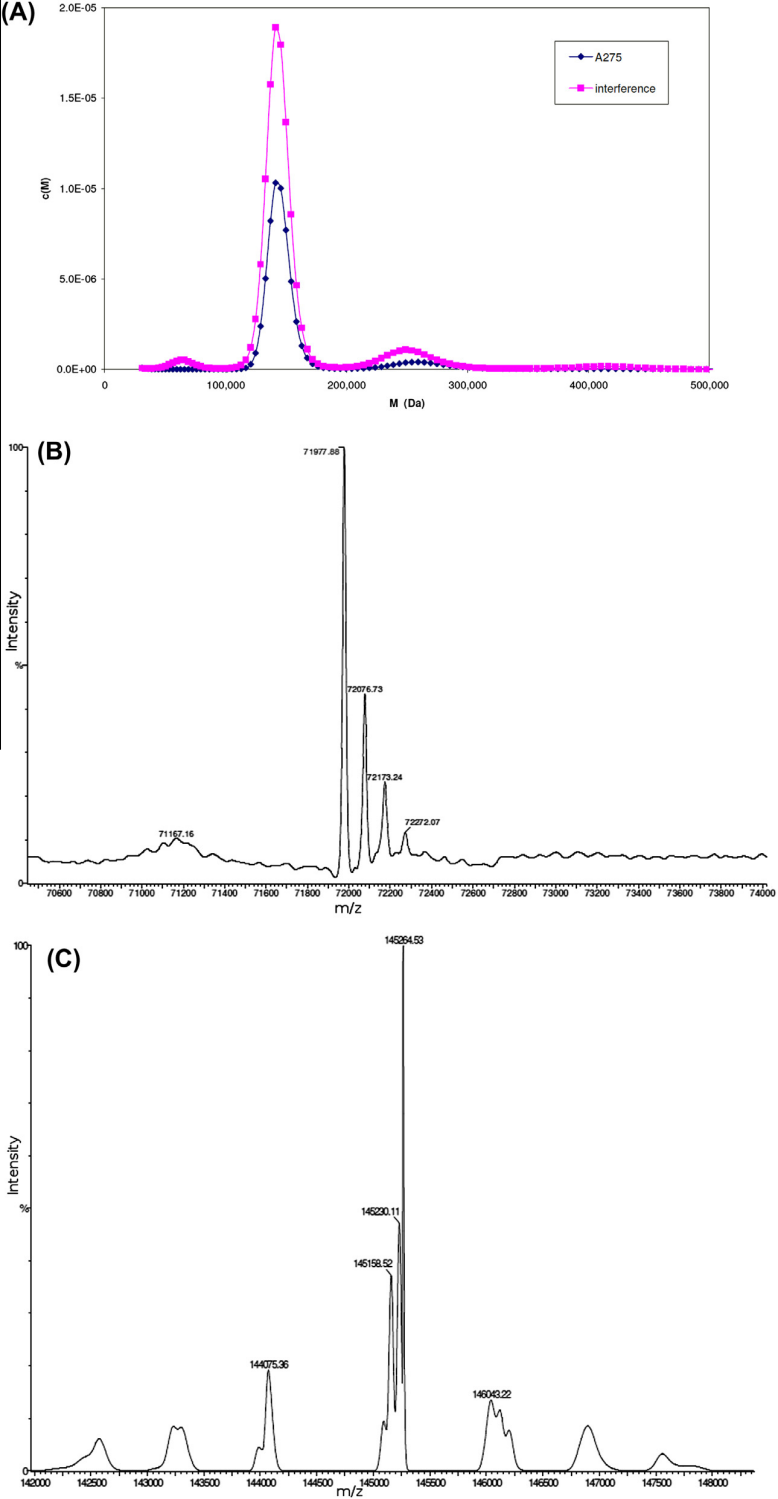
(A) Sedimentation velocity analytical ultracentrifugation experiment. APP‐1 centrifuged at 40 K rpm and protein sedimentation observed by*A*_275_ (purple) and Rayleigh interference (pink). Plot of molecular mass (Da) against the concentration per Dalton c(M) such that after integration area under each peak is equal to the loading concentration of that species. The three main species are 64,066 Da (2.1% of total), 143,268 Da (77.5%) and 250,789 Da (9.6%). (B) Electrospray mass spectrometry of APP‐1 under acidic conditions. Spectrum shows a single peak at 71977.2 Da, which fits well with the predicted mass of His‐tagged APP‐1 monomer. (C) Electrospray mass spectrometry of APP‐1 under neutral conditions. Spectrum shows a peak at 144718.6 Da, which agrees with the expected mass of a dimer.

A sedimentation velocity experiment was carried out to establish the oligomerisation state of the recombinant*C. elegans* APP‐1. Three species are evident from a plot of the molar mass (Da) against their relative concentrations (M) (Fig. 1A). Molecular masses of these species were 64,066 Da, 143,268 Da and 250,789 Da, corresponding to 2.1%, 77.5% and 9.6% of the total protein respectively. Whilst the major species is clearly a dimer, detectable amounts of monomer and tetramer are also present.

### 2.2 Mass spectrometry

APP‐1 (0.3 ml at 1.3 mg/ml) was desalted by dialysis against Millipore water for electrospray mass spectrometry. Under acidic conditions a major peak was observed at 71977.2 Da (Fig. 1B), which corresponds well with the mass of the His‐tagged APP‐1 after removal of the start methionine (calculated mass 71975.5 Da). The protein was also analysed under neutral conditions (pH 7.0) to see whether any oligomerisation could be observed. The spectrum showed a peak at 144718.6 Da indicative of a dimer (Fig. 1C).

### 2.3 Inductively coupled plasma mass spectrometry

Purified recombinant APP‐1 was dialyzed against chelex treated water to remove contaminating metal ions. A sample of protein and a control sample of buffer were subjected to metal ion analysis by ICP‐MS. The protein was quantified at 1.43 mg/ml, or 19.5 μM compared to a Zn^2+^ concentration of 21.6 μM, indicating a protein to zinc ratio of 1:1.1, with only negligible amounts of manganese and cobalt.

### 2.4 Activity of recombinant APP‐1

Purified APP‐1 converted bradykinin to des‐Arg‐bradykinin at a rate of 107 pmol/h/ng of protein and this activity was inhibited by 10 μM apstatin (92% inhibition). The enzymatic properties of the enzyme have been described previously[18].

### 2.5 Quality of the structure of*C. elegans* APP‐1

**Figure 2:**
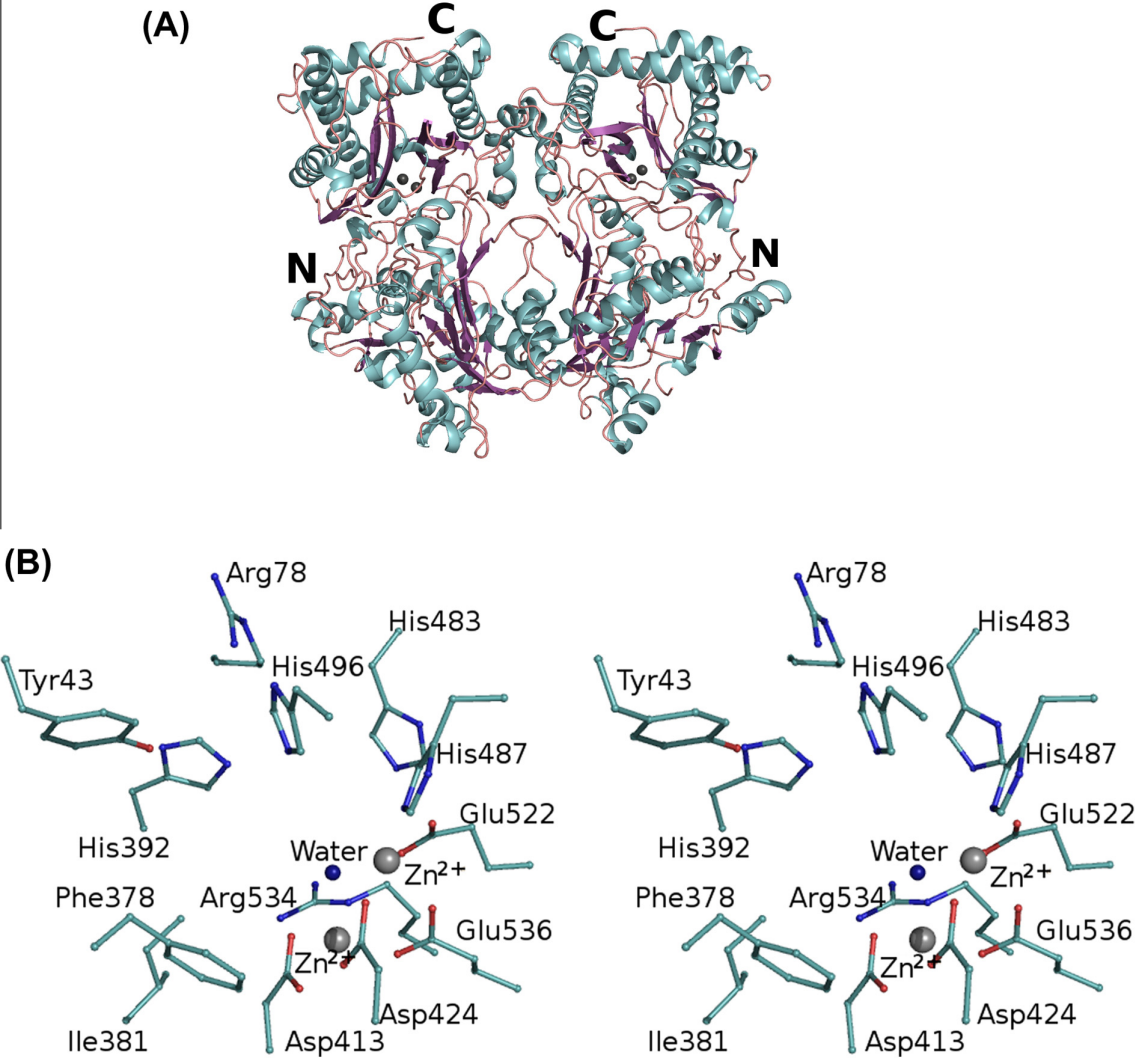
(A) Ribbon representation of*C. elegans* APP‐1. The secondary structure elements are coloured green‐cyan (β‐strands) and purple (α‐helices). The Zn^2+^ ions are represented as spheres in grey. The N‐ and C‐terminal ends of both monomers have been labelled. (B) Stereoview of the active site in native*C. elegans* APP‐1 structure. The interacting residues are coloured according to their elements: carbon, cyan; nitrogen, blue; oxygen, red. The Zn^2+^ ions are shown in grey. Active site water molecule is shown in dark blue.

The structure of*C. elegans* APP‐1 was determined at 1.93Å resolution (Fig. 2A andTable 1). The crystals contained two molecules in the asymmetric unit, each comprising the full complement of the APP‐1 amino acid sequence. Analysis of the Ramachandran plot[35] indicated that 97% of residues fall in the most favoured region with no residues in the disallowed region of the plot. Although both the polypeptide chains are complete with the exception of N‐terminal His_6_ tag, residues 69, 93, 101, 133, 158, 159, 234, 280, 394, 396, 398, 437, 528 and 598 from chain P (Mol_A_) and residues 69, 133, 159, 234, 280, 394, 396, 398, 437 and 616 from chain Q (Mol_B_) have patchy electron density for their side chains. Residues 505–508 that form part of a solvent exposed loop region have not been modelled in the structure as electron density for this region could not be observed. None of these residues form the dimer interface and thus do not affect our analysis of the interface. Average temperature factors of 32.1 Å^2^ for Mol_A_ and 31.8 Å^2^ for Mol_B_ were calculated. A total of 597 water molecules and four Zn^2+^ ions were also identified in the asymmetric unit, with an average B‐factor of 34.6 Å^2^ and 27.8 Å^2^, respectively. The atomic coordinates and structure factors for both the unliganded APP‐1 and apstatin‐bound APP‐1 have been deposited in the Protein Data Bank with accession codes4S2R and4S2T.

**Table Table 1:** Crystallographic data and refinement analysis

	Native*C. elegans* APP‐1	*C. elegans* APP‐1·apstatin complex
Space group	C2	C2
Unit cell	*a*= 141.4 Å,*b*= 87.9 Å,*c*= 114.7 Å	*a*= 140.6 Å,*b*= 86.8 Å,*c*= 113.1 Å
	*β*= 115.9°	*β*= 115.9°
Resolution range (Å)	30–1.93 (1.98–1.93)	30–2.15 (2.21–2.15)
Redundancy	3.7 (2.9)	2.6 (1.8)
*I*/*σI*	14.9 (1.9)	13.3 (2.4)
Completeness (%)	97.2 (82.7)	91.8 (60.4)
*R_p.i.m_* (all*I*+ and*I*−)	0.044 (0.408)	0.045 (0.328)
No. of reflections
Total	339,579 (16,893)	156,269 (5,362)
Unique	91,904 (5,753)	61,133 (2,969)
Wilson*B* (Å^2^)	26.3	44.3
*R*_work_ (%)	16.3	20.4
*R*_free_ (%)	21.4	24.7
Average*B* values (Å^2^)
Protein	31.9	35.6
Solvent	34.6	32.5
Ligand	–	40.1
Ions	27.8 (Zn^2+^ ions)	49.9 (Zn^2+^ ions)
		24.3 (SO_4_^2−^ ions)
Root mean square deviation
Bond lengths (Å)	0.007	0.008
Bond angles (°)	1.03	1.217

Values in the parentheses refer to the highest resolution shell.

### 2.6 Overall structure of*C. elegans* APP‐1

The final refined structure of*C. elegans* APP‐1 (Fig. 2A) is topologically similar to the structure of human X‐prolyl aminopeptidase. The*C. elegans* APP‐1 structure can be divided into three domains: an N‐terminal domain comprising residues 1–163, a middle domain consisting of residues 164–318 and the C‐terminal domain consisting of residues 319–616. The program DSSP[36] was used to assign secondary structure elements in each domain. The N‐terminal domain and the middle domain are structurally similar to one another. The core of these domains is composed of a six‐stranded β‐sheet flanked by the same number of α‐helices. All the β‐strands in each of the two domains point in the same direction except strand 2 of each domain, which points in the opposite direction. The C‐terminal domain also consists of 6 α‐helices. These helices flank the 9 β‐strands of this domain, 5 of which form an anti‐parallel β‐sheet.

### 2.7 Dimer interface

The dimeric state of recombinant*C. elegans* APP‐1 was confirmed by solution studies, including AUC and mass spectrometry (Fig. 1B and C). This is consistent with the homodimer observed in the asymmetric unit. The two protomers (Mol_A_ and Mol_B_) are held together mainly via hydrophobic interactions. Interfacing residues run along the entire length of the dimer with each subunit contributing 54 residues to the dimer interface. Around 7.5% (∼2000 Å^2^) of solvent accessible surface area from each molecule is buried upon dimer formation, calculated using the PISA interface server (http://www.ebi.ac.uk/msd‐srv/prot_int/pistart.html). There are 9 potential hydrogen bonds that help stabilize the interaction between the two subunits, of which one interaction is a salt bridge between Nζ of Lys136 and Oε2 of Glu301. The buried interface at the APP‐1 dimer is of considerable size and contains approximately one polar interaction per 220 Å^2^.

### 2.8 Active site

The catalytic site of APP‐1 (Fig. 2B) is located in a dipped surface on the five‐stranded anti‐parallel β‐sheet of the C‐terminal domain. The active site consists of residues Asp413, Asp424, His487, His496, Glu522 and Glu536, all of which coordinate with the dinuclear Zn^2+^ centre. The two catalytic Zn^2+^ ions differ in their geometry, in that one Zn^2+^ ion displays octahedral coordination geometry by interacting with all the residues listed above. The second Zn^2+^ ion on the other hand shows a distorted square‐pyramidal geometry where a water molecule and four carboxylate atoms from Asp413, Asp424, Glu522 and Glu536 form the donor atoms.

### 2.9 Structure of the*C. elegans* APP‐1·apstatin complex

**Figure 3:**
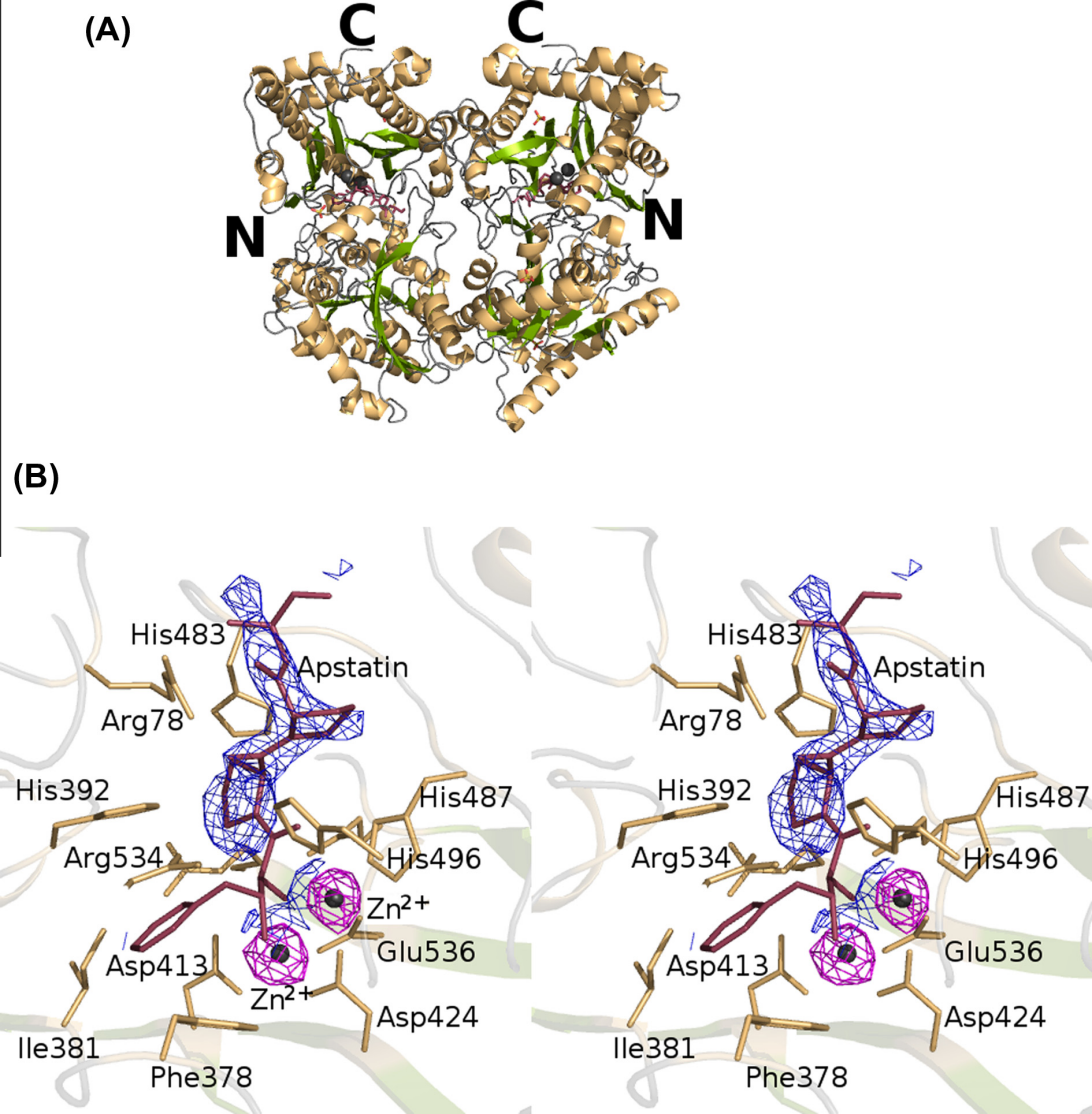
(A) Ribbon representation of*C. elegans* APP‐1 in complex with apstatin. The secondary structure elements are coloured green (β‐strands) and orange (α‐helices). The Zn^2+^ ions are represented as spheres in grey. The N‐ and C‐terminal ends of both monomers have been labelled. Apstatin is shown at the active site as a ball‐and‐stick model in raspberry red. (B) Stereoview of the active site in the complex of*C. elegans* APP‐1 with apstatin. The interacting residues are coloured orange. The inhibitor, apstatin, coloured as above is shown in ball‐and‐stick. Fo‐Fc omit map for the bound inhibitor is shown in grey. The Zn^2+^ ions are shown in cyan with the electron density shown in magenta, contoured at 3σ.

The complex of*C. elegans* APP‐1 with apstatin was determined at 2.15 Å resolution (Fig. 3A). The structure consists of two molecules in the asymmetric unit, with four Zn^2+^ ions (two per monomer) and one molecule of apstatin per molecule of APP‐1. Both the polypeptide chains are complete with the exception of N‐terminal His_6_ tag. However, residues 69, 93, 101, 133, 158, 159, 274, 280, 394, 396, 398, 437, 528 and 607 from chain P (Mol_A_) and residues 24, 69, 101, 133, 159, 192, 280, 394, 396, 398, 437, 528 and 604 from chain Q (Mol_B_) show very patchy electron density for their side chains. Similar to the native APP‐1 structure, the residues of the solvent exposed loop, 505–508, have not been modelled in the structure due to lack of electron density. A total of 355 water molecules along with seven SO_4_^2‐^ions were modelled in the structure.

Weak electron density was identified for apstatin in both the subunits in the asymmetric unit of crystalline*C. elegans* APP‐1 (Fig. 3B). Apstatin was modelled at the active site based on the*E. coli* structure using this electron density as a guide. The binding of apstatin does not cause any major structural change. The backbone of the apstatin‐bound*C. elegans* APP‐1 superposes on the C^α^ atoms of the native structure with an r.m.s.d of 0.3 Å. The inhibitor, sitting at the bottom of the active‐site cleft, makes 13 potential hydrogen‐bonding interactions with the enzyme and one bond with a water molecule (Table 2). The amino acid residues that contribute to the bonding interactions between the inhibitor and the enzyme are Arg78, Phe378, His392, Asp413, Asp424, His487, His496, Glu522 and Glu536. Apstatin displaces a water molecule present in native*C. elegans* APP‐1 to coordinate with the metal ions in the dinuclear active site. The inhibitor also makes a number of putative van der Waals interactions with the residues of*C. elegans* APP‐1 (Table 2). The phenyl ring of the first residue of apstatin (pseudo‐Phe), by itself, makes several such van der Waals contacts. The ring fits between His392 and Phe378, making loose stacking‐interactions with the two rings on either side of it. The second residue of the apstatin makes contact with Arg78, His392, His483, Glu522 and Arg534. The prolidyl ring of the third apstatin residue interacts with all the active site residues as well as Phe378 and His392 (Fig. 3B). The two C‐terminal residues of the inhibitor point towards the solvent exposed loop comprising residues 505–509. However, since these residues have not been modelled in the structure, no potential hydrogen bonding interactions or van der Waals contacts could be observed.

**Table Table 2:** Potential molecular interactions between APP‐1 residues and apstatin. Interactions were identified using CONTACT in CCP4[52]

APP‐1 residues	van der Waals contacts	Hydrogen bonds
		Apstatin	APP‐1	Distance (Å)
Tyr43	11	OBE	WATER	2.8
Arg78	10	OAXO	NH1	3.0
			NH1	2.5
Phe378	10	–	–	–
Ile381	3	–	–	–
His392	15	O	NE2	3.1
Asp413	6	OAD	OD2	3.3
		NAC	OD1	3.2
		NAC	OD2	2.9
Asp424		–	–	–
His483	6	–	–	–
Gly484	4	–	–	–
Gly486	1	–	–	–
His487	2	OAM	NE2	3.0
His496	6	OAM	NE2	2.5
Glu522	10	OAD	OE1	2.7
		N	OE1	3.06
		OAM	OE2	3.2
Arg534	4	–	–	–
Glu536	2	OAD	OE1	3.3
		OAD	OE2	3.2

## 3 Discussion

### 3.1 Metal ion dependence of*C. elegans* APP‐1

The metal ion content of purified recombinant His‐tagged APP‐1 was analysed by ICP‐MS, which revealed a 1:1.1 M ratio of protein to Zn^2+^ with only negligible quantities of Mn^2+^ and Co^2+^. The near 1:1 ratio is consistent with inductively coupled plasma atomic emission spectroscopy (ICP‐AES) results for a number of purified clan MG enzymes which showed the presence of ∼1 M equivalent metal ion. These included 1 M equivalent Co^2+^ in*E. coli* MAP[37], one Zn^2+^ in*Pyrococcus furiosus* MAP[38], one Co^2+^ in recombinant*P. furiosus* prolidase[39], one Zn^2+^ in purified porcine APP‐2[40] and one Mn^2+^ ion in recombinant human APP‐1[41]. However, the metal content and metal‐protein stoichiometry can be influenced by the cell culture conditions employed to express the recombinant protein and especially whether the medium has been enriched with a particular metal. For example, the human APP‐1 used for structural studies was expressed in*E. coli* cultured in Mn^2+^‐enriched LB medium under slow growth conditions contained 1.79 Mn^2+^ ions per protein monomer, but when expressed in plain LB medium the metal ion content changed to a mixture of Mg^2+^, Mn^2+^ and Fe^2+^ in a ratio of 0.41, 0.70 and 0.73[29]. Nevertheless the molar ratio of total divalent metal to APP‐1 monomer remained close to 2:1. This ratio is consistent with the results from several crystallographic studies of clan MG peptidases, including data presented here for*C. elegans* APP‐1 that reveal two metal ion binding sites in the active site, and most proposed mechanisms for these enzymes predict the nucleophile to be a bridging water (or hydroxide) ligand between these two metals. Whilst some clan MG peptidases, such as*E. coli* APP[25] have been crystallized with a single non‐catalytic metal ion in the active site, it was only recently that the crystal structure of a “mono‐metalated” MAP structure was published containing a single catalytically relevant manganese ion[42]. This was achieved by tightly regulating the concentration of various metal ions within the crystallization solution. As mentioned above, ICP‐AES and ICP‐MS identified Mn^2+^ as the divalent cofactor in human APP‐1. However ICP‐MS and crystallographic analysis of*C. elegans* cytosolic APP‐1 has shown the presence of Zn^2+^, with only negligible quantities of Mn^2+^ and Co^2+^. Interestingly, mammalian membrane‐bound APP‐2 is also a zinc metalloenzyme[13].

### 3.2 C. elegans APP‐1 forms a dimer

Recombinant His‐tagged*C. elegans* APP‐1 forms a dimer. Electrospray mass spectrometry under neutral conditions showed two peaks, one corresponding to the 72 kDa monomer and the second with a mass 144 kDa, presumably corresponding to a dimer. To confirm that APP‐1 forms a dimer in solution a sedimentation velocity analytical ultracentrifugation experiment was performed which showed that 80% of the total protein was present as a species with a mass of 143 kDa (Fig. 1A). The protein also crystallizes as a dimer in the asymmetric unit, in both native and inhibitor bound states (2, 3A and 3A).

Unlike the recombinant His‐tagged*C. elegans* APP‐1, the*E. coli* homologue forms a tetramer where two monomers form a dimer through interactions between the C‐terminal domains, and then these two dimers interact through their N‐terminal domains[26]. Therefore the possibility that the N‐terminal His‐tag and linker are disrupting formation of a tetramer in the His‐tagged*C. elegans* APP‐1 cannot be excluded. However, analysis of the oligomerisation state of other animal cytosolic APP‐1 homologues by various size exclusion techniques suggest that it is a common structural feature of these enzymes to form dimers in solution at physiologically relevant salt concentrations[41, 43, 44]. Rat brain APP‐1, known to form a dimer in the presence of ∼140 mM salt, forms trimers in the absence of NaCl[22]. Whilst cytosolic APP‐1 forms dimers, the membrane bound APP‐2 appears to differ with a range of oligomerisation states depending on the species the enzyme is from. For example, bovine lung APP‐2 forms a tetramer[45], rat lung APP‐2, varies between a trimer and a tetramer depending on NaCl concentration[46] and pig kidney APP‐2 forms a trimer[40]. The physiological relevance of this difference between APP‐1 and APP‐2 is unclear.

### 3.3 Comparison with human X‐prolyl aminopeptidase

**Figure 4:**
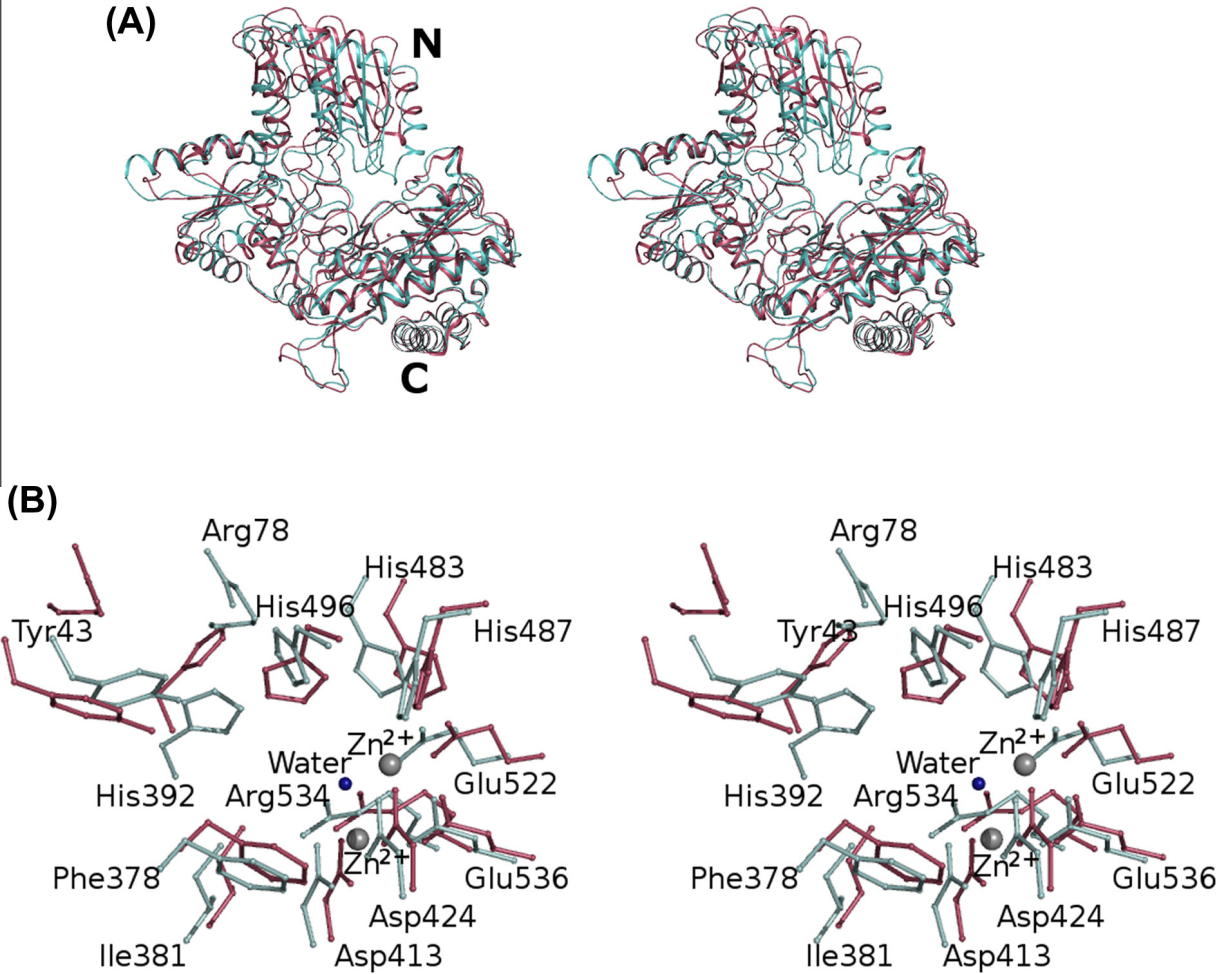
Stereo superposition of the C^α^ traces of*C. elegans* APP‐1 and human APP‐1. (A) Human APP‐1 is shown in raspberry red and the*C. elegans* APP‐1 is shown in palecyan. N‐ and C‐termini are labelled. (B) Stereo superposition of the residues at the active site. Colours correspond as above. Residues from the*C. elegans* APP‐1 structure are labelled.

Human APP‐1 structure, when elucidated, revealed for the first time a novel three‐domain structure for an X‐prolyl peptidase[29]. Comparing this modular arrangement with that of*C. elegans* APP‐1 it becomes obvious that the three domains in our structure occupy similar statutory positions (Fig. 4A).*C. elegans* APP‐1 is, however, the first structure of a eukaryotic APP‐1 with an inhibitor molecule bound at the active site. The human APP‐1 structure solved previously is a native structure. Like*C. elegans* APP‐1, human APP‐1 is around 620 amino acids long, but despite topological similarities, a lot of conformational differences can be observed when the two structures are compared. The most significant of the differences can be seen in the N‐terminal region of the structures (Fig. 4A). Superposition of the human enzyme on to that of*C. elegans* leads to an r.m.s.d. of ∼2.1 Å over 551 C^α^ atoms. The structural correspondence between the two increases, with an r.m.s.d. of 1.1 Å over 433 C^α^ atoms, when the structures are superposed after the removal of the N‐terminal domain. There are also some significant differences seen in the positioning of the catalytically important residues, especially the residue corresponding to His392, which seems further away from the active site in human APP‐1 (Fig. 4B). Also, residue Arg77 in human APP‐1 (Arg78 in*C. elegans* APP‐1) is further away from the active site. One must, however, bear in mind that the human APP‐1 structure does not have apstatin bound at the active site. The orientations and positioning of these side chains in the structure of the human enzyme might be different when bound to apstatin.

### 3.4 Comparison between*C. elegans* APP‐1·apstatin structure and*E. coli* APP‐1·apstatin structure

**Figure 5:**
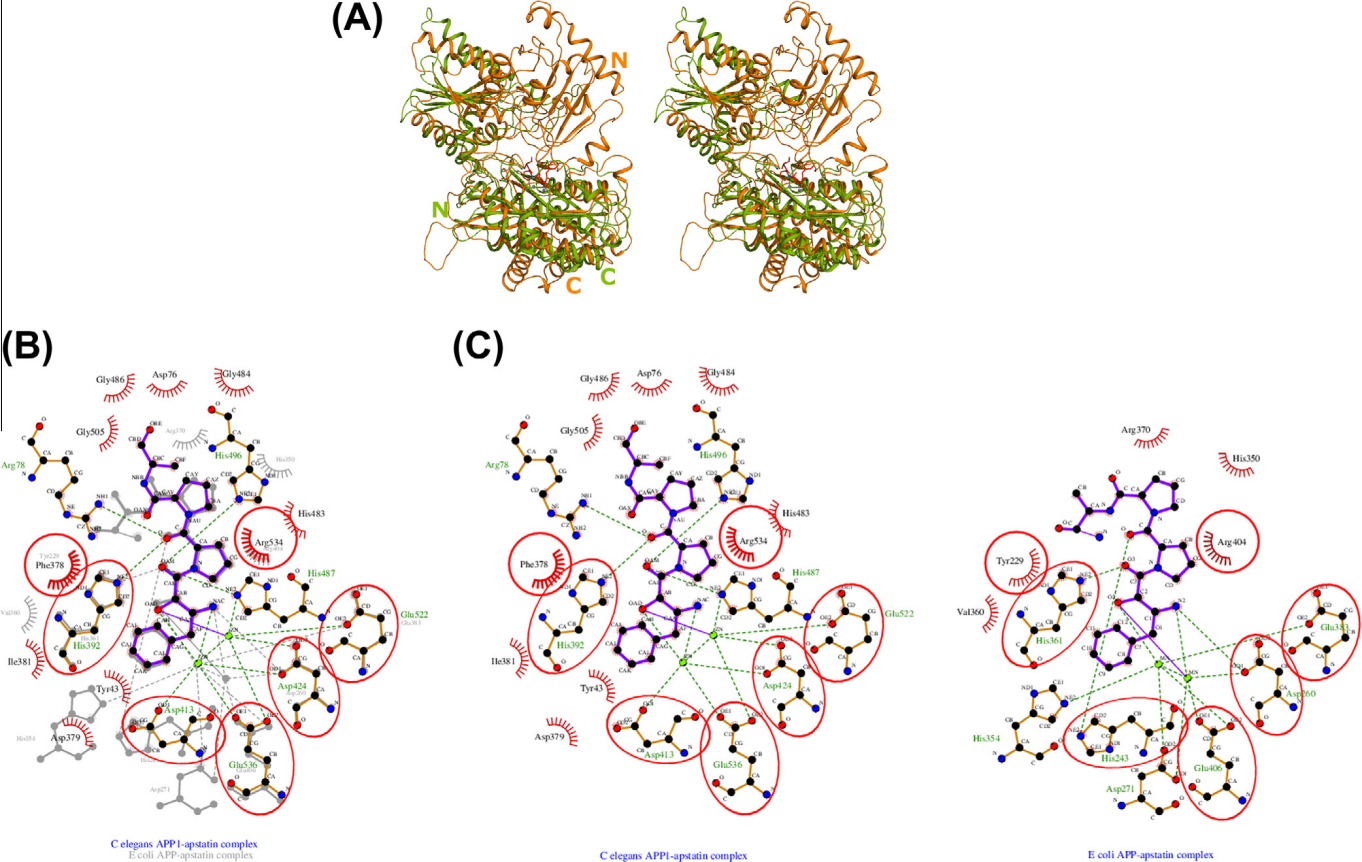
Comparison of*C. elegans* APP‐1·apstatin and*E. coli*·APP‐1. (A) Stereo superposition of the C^α^ traces of*C. elegans* APP‐1·apstatin and*E. coli*·APP‐1.*E. coli*·APP‐1 is shown in splitpea green and the*C. elegans* APP‐1·apstatin is shown in orange. N‐ and C‐termini are labelled. (B) Superposition of the schematic 2D representation of the molecular interactions between APP‐1 and the ligand in the two apstatin complexes. (C) Schematic 2D representation of the molecular interactions between APP‐1 and the ligand in both complexes.

Structure superposition of*C. elegans* APP‐1 and*E. coli* APP‐1[27] shows that even though r.m.s. difference between the positions of corresponding atoms is 2.6 Å, the two share a conserved catalytic domain and active site (Fig. 5A). Superposition of the active sites from the two complexes (Fig. 5B) reveals a close correspondence between the coordination geometries and the conformation of the residues surrounding the two metal ions at the dinuclear active site.*E. coli* APP‐1 requires Mn^2+^ for its activity, whereas*C. elegans* APP‐1 has Zn^2+^ as the active site metal. We used LIGPLOT[47] to generate schematic 2D representation of the putative molecular interactions between APP‐1 and the ligand in the two apstatin complexes to simplify visualisation of the active site.

The catalytic mechanism of*E. coli* APP‐1 has been extensively studied[25, 48]. Given the close agreement between the active sites, it is likely they share the same enzymatic mechanism. In the*E. coli* structure, the catalytic His243 is thought to participate in hydrogen bonding interaction with the proline residue of the substrate to stabilise its binding at the active site (Fig. 5C). Mutation of this residue to alanine leads to a significant decrease in the catalytic efficiency of the*E. coli* enzyme. Based on the fact that this histidine is conserved across all X‐prolyl aminopeptidases, there is little doubt that His392 plays a similar important role in*C. elegans* APP‐1. In the present complex structure, this residue makes 15 potential van der Waals contacts at the active site along with one potential hydrogen‐bonding interaction (Fig. 5C). Also seen in the present structure is the side chain of Arg78 pointing towards the active site, forming two hydrogen bonds with the inhibitor molecule (Fig. 5C). Arg78 probably helps to hold the inhibitor in place in the active site by means of this hydrogen bond. The equivalent residue is however missing in*E. coli* APP‐1 due to the lack of the N‐terminal domain (domain I). Even though the general positioning of the inhibitor molecule at the active site is similar in both our complex and the*E. coli* APP·apstatin complex (Fig. 5B), the position of the second, specificity‐conferring prolidyl ring in our complex is such that it makes contacts with the enzyme at Arg78 as well as His487 in addition to its interactions with five other APP‐1 residues (Phe378, His392, His496, Arg534) and the metal‐coordinating residue, Glu522 via the active site Zn^2+^ ions. Counterpart residues in the*E. coli* APP·apstatin complex are Tyr229, His350, His361, Arg404 and Glu383, respectively[27]. The two complexes also show the C‐terminal residues protruding in two opposite direction from their respective active sites (Fig. 5B). But since both complexes did not show sufficiently resolved density of the last two apstatin residues, it is entirely possible that the orientation and positioning might be the same in the two complexes. .

## 4 Conclusions

The striking similarity between the structures of X‐prolyl aminopeptidases reveals similar polypeptide topologies and almost identical active sites, suggesting that these enzymes also share similar modes of substrate binding and a common catalytic mechanism. In this paper we have presented the structure of*C. elegans* APP‐1 both in its native form as well as in complex with the inhibitor, apstatin. Comparison of all published X‐prolyl aminopeptidase structures reveals that the residues that have been shown to interact with apstatin in one enzyme are either strictly or conservatively conserved in the others. It can be assumed that the interactions observed in the complexes of apstatin with*E. coli* APP‐1 and*C. elegans* APP‐1 would be replicated to some extent in the complex of human APP‐1 with apstatin. Although it is still not possible to explain the absolute requirement of a penultimate N‐terminal proline residue in substrates, the structure of the modelled apstatin‐*C. elegans* APP‐1 complex provides valuable insights into the specificity of the enzyme. The structure provides a scaffold for the design of inhibitors that are specific for*C. elegans* APP‐1 by taking advantage of the structural differences and similarities between the different enzyme‐inhibitor complexes.

## 5 Materials and methods

### 5.1 Cloning, expression, and purification of recombinant*C. elegans* APP‐1

*C. elegans* APP‐1 cDNA was PCR‐amplified using Expand High Fidelity Enzyme (Roche) and the following primers: forward primer 5′‐CATATGACAGCATTGGAAAAACTC‐3′ and reverse primer 5′‐CATATGTTAGATTGGTTTACAGGC‐3′. The PCR product was cloned into pET19b plasmid (Novagen), and the resulting expression plasmid was transformed into*E. coli* BL21 star (DE3) strain (Stratagene).

Cells harbouring the pET19b‐APP‐1 expression plasmid were grown in autoinduction media[49] at 25 °C and harvested 40 h post inoculation. Cells obtained from 0.5 L of bacterial culture were resuspended in 20 ml of lysis buffer containing 10 mM Tris/HCl, 0.3 M NaCl at pH 8 supplemented with 20 mM imidazole. Cells were lysed using a cell disruptor at 20,000 PSI. The lysate was centrifuged for 30 min at 70,000×*g* at 4 °C to remove cellular debris. The supernatant was loaded onto a 5‐ml HiTrap FF column (GE Healthcare). The column was washed with lysis buffer to remove unbound material, following which the bound protein was eluted using 0.3 M imidazole in lysis buffer. The eluate from the HiTrap FF column was concentrated and further purified on Superdex 200 (GE Healthcare), equilibrated with 10 mM Tris/HCl, pH 8.0, and 150 mM NaCl. Purity and homogeneity of the protein were monitored by SDS–PAGE at every step. APP‐1 was concentrated to 3 mg/ml for the purpose of crystallization. The purified protein was authenticated by mass spectrometry and western blot analysis.

### 5.2 Protein crystallization, data collection, and processing

Crystals were grown using the sitting drop vapour diffusion method at 16 °C. Crystallization buffer containing 17.5% PEG 3350, 0.1 M Bis‐tris propane, pH 8.5, and 0.2 M sodium malonate was mixed with an equal volume of protein solution. A complete dataset to 1.9 Å resolution was collected from a single crystal using the Diamond Light Source, UK. Crystals for the complex of*C. elegans* APP‐1 with apstatin were obtained by overnight incubation of the protein at 3 mg/ml with 10 mM inhibitor at 4 °C prior to setting up crystallizations. Crystals of the complex grew at the pH 7.5 in 20% PEG3350. A complete dataset to 2.15 Å resolution was collected from a single crystal using the Diamond Light Source, UK. Data were processed and scaled using HKL2000[50] in the monoclinic space group C2.

### 5.3 Structure determination and refinement

The structure of*C. elegans* APP‐1 was determined by maximum likelihood molecular replacement using the program PHASER from CCP4 suite[51, 52]. The initial search model used was human cytosolic APP‐1 (PDB: 3CTZ). The asymmetric unit consists of an APP‐1 dimer. Iterative rounds of model building with the program COOT[53] and refinement with the program PHENIX[54] resulted in the final native APP‐1 structure for all data between 40 and 1.9 Å resolution. The structure of*C. elegans* APP‐1 in complex with apstatin was determined using the native structure solved above. The asymmetric unit consists of two molecules of APP‐1. Iterative rounds of model building with the program COOT[53] and refinement with the programs PHENIX[54] in the early stages and REFMAC from CCP4 suite[52, 55] in the later stages resulted in the final APP‐1·apstatin complex structure for all data between 40 and 2.15 Å resolution. Structure validation was carried out using MOLPROBITY[56, 57] and the RCSB PDB Validation Suite (http://deposit.rcsb.org/validate/). Refinement statistics for the finalised structures are provided inTable 1. Figures were prepared using PYMOL (http://www.pymol.org). The atomic coordinates and structure factors for native*C. elegans* APP‐1 (PDB: 4S2R) and its complex with apstatin (PDB: 4S2T) have been deposited with the Protein Data Bank, Research Collaboratory for Structural Bioinformatics, Rutgers University, New Brunswick, NJ, USA (http://www.rcsb.org/).

### 5.4 Analytical ultracentrifugation

1 ml of 0.3 mg/ml APP‐1 was dialyzed against 50 ml 10 mM Tris/HCl pH 7.5, 150 mM KCl overnight. Both protein and a reference sample of the dialysis buffer were submitted to to the Astbury Centre for Biomolecular Interactions, University of Leeds, for ultracentrifugation. Samples (0.4 ml) were centrifuged in 1.2 cm path‐length 2‐sector aluminium centre‐piece cells (sample in RH sector, reference buffer in LH sector) with sapphire windows in a 4‐place An‐60 Ti analytical rotor running in an Optima XL‐I analytical ultracentrifuge (Beckman Instruments, Inc., Palo Alto, California 94304) at 40,000 rpm and at a temperature of 20 °C. Changes in solute concentration were detected by Rayleigh interference and 275 nm absorbance scans. 153 scans were recorded during 3 h 40 min.

### 5.5 Mass spectrometry

0.3 ml of APP‐1 at 1.3 mg/ml was dialyzed against Millipore water in a 3 kDa MWCO dialysis tubing (Spectropore). The dialyzed protein sample was subjected to electrospray mass spectrometry under acidic and neutral conditions at the Astbury Centre, University of Leeds.

### 5.6 Inductively coupled plasma mass spectrometry (ICP‐MS)

APP‐1 (4 ml of 1.43 mg/ml) was dialyzed against chelex treated Millipore water in dialysis tubing that had been EDTA treated to remove contaminating metal ions. The protein and a control sample of the dialysis buffer were submitted for metal ion analysis by ICP‐MS using Agilent Technologies 4500 (School of Analytical Sciences, University of Sheffield). Instrumental calibration was achieved through the use of a single multi‐element standard solution (10 μg/ml).

### 5.7 APP activity assay

Enzyme activity was assayed by HPLC using bradykinin (40 μM; Arg‐Pro‐Pro‐Gly‐Phe‐Ser‐Pro‐Phe‐Arg) as the substrate and UV absorbance (214 nm) to quantify the reaction product, des‐Arg‐bradykinin[18]. Reactions were carried out in 50 μl of 0.1 M Tris/HCl, pH 8.0 at 25 °C with 6 ng of recombinant protein and were terminated by the addition of 10 μl of 8% trifluoroacetic acid. RP‐HPLC was performed using a 25‐cm × 0.45 mm C_18_, 5 μm column (Phenomenex, Macclesfield, Cheshire, England) and a solvent gradient ranging from 18% to 70% (v/v) acetonitrile in 0.1% trifluoroacetic acid over 20 min and flow rate of 1 ml min^−1^. Inhibition of APP‐1 activity by 10 μM apstatin was performed by pre‐incubating inhibitor with enzyme for 10 min prior to starting the reaction by the addition of substrate.

## 6 Author contribution

SI performed protein expression, purification, structural biology experiments, analysed the structures and wrote the manuscript. PL‐B performed protein expression, purification and initial crystallisation studies. KAPP and MRP performed preliminary analysis of X‐ray diffraction data and biophysical experiments. REI and AJT supervised the biophysical study and edited the manuscript. KRA conceived the study, supervised the structural study, analysed the data, wrote and edited the manuscript. All authors reviewed the manuscript.
